# Formative experiences and psychological distance in the lives of contemporary environmentalists

**DOI:** 10.3389/fpsyg.2023.1192018

**Published:** 2023-07-18

**Authors:** Urooj S. Raja, Amanda R. Carrico

**Affiliations:** ^1^School of Communication, Loyola University Chicago, Chicago, IL, United States; ^2^Department of Environmental Studies, University of Colorado Boulder, Boulder, CO, United States

**Keywords:** climate change, environmentalists, significant life experiences (SLE), psychological distance (PD), public engagement on climate change, climate activism

## Abstract

**Introduction:**

We use the term “environmentalists” to describe the people who are highly and actively engaged and involved in environmental issues like climate change. Environmentalists consistently advocate, research, or volunteer to do the work needed to address environmental challenges. Factors that drive contemporary environmentalists remain understudied.

**Methods:**

We, therefore, ask: what formative experiences drive environmentalists on climate change and other environmental problems at present? We frame this exploration through the significant life experiences (SLE) literature, which examines people and environmental pathways. We also ask: how do feelings of perceptual/actual distance or closeness to environmental threats and climate change shape a person’s decision to become an environmentalist? We anchor this query to the psychological distance (PD) literature that explores how people perceive external phenomena and the role distance plays in their conception. To answer both questions, we use qualitative methods and draw on 33 interviews with environmentalists involved in environmental protection work for an average of 91 h in the past 4 weeks.

**Results:**

We find that environmentalists spoke about several formative experiences broadly consistent with what has been documented in the SLE literature. Traumatic experiences were especially influential for our sample of environmentalists. Our findings also reveal that PD, especially social, plays a role in the lives of environmentalists.

**Discussion:**

Study findings could help scholars and practitioners deepen their understanding of contemporary environmentalists. Practitioners, in particular, could use this knowledge to help environmentalists by providing them with tailored resources and support.

## 1. Introduction

We use the term “environmentalists” to describe the people who are highly engaged and active in ameliorating environmental issues. Environmentalists consistently advocate, research, or volunteer to do the work needed to address environmental challenges like climate change. Environmentalists like Rachel Carson, Wangari Maathai, and Greta Thunberg have significantly contributed to raising awareness and promoting action about environmental issues like climate change. Climate activists, in particular, may also help drive decision-making (Fisher, 2019; Fisher and Jorgenson, 2019; Fisher and Nasrin, 2021). Environmentalists are also an important group to study, given they often generate the much-needed momentum to drive change on worsening climate change (IPCC, 2018; Masson-Delmotte et al., 2021; Pidcock et al., 2021) and persisting political gridlock on climate change (Nisbet, 2009; Whitmarsh et al., 2013; Tyson et al., 2021).

Moreover, by some estimates, nearly half the American public may be motivated to do something about climate change. For example, in the latest Yale Program on Climate Change Communication survey from December 2022, Anthony Leiserowitz and colleagues examined six different interest categories of the American public (*n* = 1,085). They discovered that approximately 53% of people were either alarmed or concerned about climate change which they describe as the most concerned and most motivated group among the six categories. This motivated group was made up of two categories: (the alarmed (26%) and the concerned (27%) (Leiserowitz et al., 2023). Considering these current engagement trends, we examine environmentalists already highly engaged in problems like climate change. By understanding the formative experiences and factors that shaped their engagement, we can perhaps gain insight into motivating greater involvement among those who may not be as involved at present but feel motivated to do more.

What we know about environmentalists or highly engaged persons comes in part from the Significant Life Experiences (SLE) literature, which, as its name suggests, describes the formative experiences or SLEs driving the highly engaged (Tanner, 1980; Hines et al., 1987; Chawla and Cushing, 2007; Matsuba and Pratt, 2013; Howell and Allen, 2019). For example, scholars within this literature speak about the importance of experiences in nature and experiences with role models who shape care for the environment. Recent work has reexamined influences to see if they still hold. One study, for example, found that specific experiences, for example, time spent in nature, may not be as formative as once believed (Howell and Allen, 2019). Consequently, we know less about whether previously named life experiences stay salient in the lives of current environmentalists. Moreover, much of the SLE research applies to engaged persons from the past who were most likely dealing with different problems than their contemporary peers.

Evidence from engagement research further suggests that perceiving environmental issues as far away, which is to say, feeling psychologically distant, is one reason for broad environmental disengagement (for overview see Trope and Liberman, 2010; Spence et al., 2012; Maiella et al., 2020). Whether and how environmentalists have overcome the psychological distance that produces apathy towards environmental problems for so many is an underexamined question. For these reasons, we ask two questions in this study: *first*, what formative experiences drove environmentalists to pursue careers or activistic work to combat environmental problems? *Second*, did psychological distance to climate change, or the lack thereof, factor into their decision to follow environmentalist engagement pathways? We use data from interviews with 33 contemporary environmentalists to answer these questions. Our environmentalists were individuals who advocate, volunteer and engage in climate change or environmental-focused work as non-profit workers, teachers, lawyers, academics, and communicators.

This article offers insight into the formative experiences of those on the frontlines of contemporary environmental challenges. It further explains how perceptions of distance from environmental problems shape environmentalists’ thoughts and feelings and whether a lack of distance motivates their decisions to engage. Most importantly, our findings may supply insights that provide a more nuanced understanding of the motivators driving contemporary environmentalists.

## 2. Conceptual framework

### 2.1. Formative experiences and environmental engagement

Emerging in the 1980s, SLE research seeks to understand what early life experiences motivate people to enter environmental protection pathways (Tanner, 1980; Payne, 1999; Chawla, 2020; Chawla and Gould, 2020; Van Heel et al., 2023) or to understand the antecedents behind broader environmental protective value and behaviors (Howell and Allen, 2017; Rosa et al., 2018). Within SLE research, engaged persons have named nature experiences as formative (Williams and Chawla, 2016; Howell and Allen, 2017; Barratt Hacking et al., 2020), which may include, for example, camping and hiking outdoors. Such experiences usually happen routinely and for prolonged amounts of time, especially in childhood (Chawla and Gould, 2020; D’Amore and Chawla, 2020). Other formative influences include interacting with role models who exhibit care and respect for the environment (especially parents and teachers). Exposure to books and media featuring strong protagonists, often interacting with and in harmony with nature (for example, *Julie of the Wolves*), are also possible influences that could lead to higher engagement in later life (Williams and Chawla, 2016). Despite their prevalence in the formation stories of those who embark on environmental vocations, nature experiences are highly subjective and may not be formative for everyone; for example, a person cleaning a park for credit (Payne, 1999).

SLE scholars mainly studied environmental educators and conservation professionals (Tanner, 1980; Chawla, 2006, 2020). Most people within these groups often had institutional affiliations with big green organizations (Chawla, 2020; D’Amore and Chawla, 2020) like the National Audubon Society (Tanner, 1980; Matsuba and Pratt, 2013) and were highly educated (Wright and Wyatt, 2008). Less attention appears to be focused on the formative experiences driving activists (Fritsche and Masson, 2021). Among the studies that do examine environmental activists, the work focuses on environmental issues of concern that were at the forefront when the SLE field began and promulgated in the 1980s and 1990s; for example, researchers in this vein have interviewed people who protested radioactive disposal at national park sites (Chawla, 2001). Less SLE work is on climate activists (Chawla, 2020; D’Amore and Chawla, 2020). Research exists on youth climate activists but appears to focus more on the outcomes of their pursuits rather than the experiences that drive them (Fisher, 2019; Fisher and Nasrin, 2021). Other research focuses on climate activists outside the United States (Fisher, 2016; Howell and Allen, 2019).

SLE work has also been criticized for focusing only on white participants, those from high social-economic statuses, and those working in prestigious environmental organizations, often full-time (Barratt Hacking et al., 2020). A more fluid and inclusive definition of a contemporary environmentalist incorporating a diversity of vocation (volunteer or work), racial diversity (environmentalists that identify as non-white), and that work on climate change is needed.

Moreover, contemporary explorations of what propelled environmentalists, especially those who work and focus on climate change and related issues, remain needed. First, given that much of the instrumental SLE work was done decades ago (Tanner, 1980; Chawla, 1999). Scholars who recognize the changing nature of environmental work further emphasize that formative influences evolve. For example, a novel contribution of Arnold et al. (2009) study: social interactions with peers, instead of parents and teachers, played a crucial role in driving environmental leader’s decision to engage; that is, the role of peers was more instrumental in 2009 than in the past for the sample under study. Scholars, in this vein, periodically evaluate the relevance of significant life experiences identified in the literature to see whether and if they hold as we do here. Second, environmental activists are no longer perceived as a community of “uninformed tree huggers” (Chawla, 2001), often as their predecessors were. The nature of environmental problems has also changed. For example, major industries like the renewable solar energy industry are premised on helping and not harming the environment (Olabi and Abdelkareem, 2022). Big business is also attached to the environment now than when the SLE literature rose to prominence. For example, according to one financial blog, the renewable energy sector is generating profit returns of almost 200% as compared to about 80% for fossil fuel companies (GreenPortfolio, 2023) and is attracting consumer attention. Consequently, what it means to be engaged today may differ from a decade or even a few years ago.

SLE scholars are also raising questions about how formative experiences in nature are at present. For example, the authors of one study found that childhood experiences in nature were generally not described as influential for a United Kingdom sample (Howell and Allen, 2019). In an earlier study, the same authors surveyed 344 individuals who adopted green behaviors (installing home insulation and reducing travel, for example). They found that exposure to nature and the outdoors was not a significant factor (Howell and Allen, 2017, 2019). Similar studies suggest we should revisit the SLE findings to see if they hold especially given the changing nature of environmental challenges.

Given that much of the SLE literature also seeks to examine what experiences could instill empathy or care for the environment in individuals, literature on disengagement on climate change may be good to bring into conversation with SLE work. Psychological distance is a key construct that scholars examine when it comes to investigating apathy and disengagement on climate change (Spence et al., 2012; Sacchi et al., 2016; Jones et al., 2017).

### 2.2. Psychological distance and environmental engagement

What is psychological distance? When you think or feel that some phenomenon is apart or disconnected from you or abstract to you, you can be said to be experiencing psychological distance (Bar-Anan et al., 2006; Trope and Liberman, 2010; Liberman and Trope, 2014). Such phenomena could be events, problems, places, or people. In some ways, psychological distance is more concerned with perceptual rather than physical distance. This thinking and feeling of perceptual and sometimes actual distance or disconnection may manifest in four ways: geographically, temporally, socially, and hypothetically. To illustrate, imagine the case of Julia from Vermont and the problem of sea level rise in Mauritius. First, Julia may feel geographically distant from sea level rise in Mauritius because she lives far away from Mauritius. Second, Julia may feel socially distant from sea level rise in Mauritius because she thinks and feels that neither she nor her family or friends are directly affected. Third, Julia may feel temporally distant because she thinks or feels that sea level rise may occur 50 years later. Finally, she may feel hypothetically distant if she is uncertain that sea level rise is occurring.

Both the SLE and PD frameworks emphasize environmental engagement. In the SLE work, environmentalists share a cluster of experiences that shaped their affinity for the environment in later life (Tanner, 1980; Chawla and Cushing, 2007; Chawla, 2020). Similarly, in the PD framework, scholars explore the obstacles responsible for inaction on climate change and find that when people think or feel that an event is abstract or removed from them in space or time, they are less likely to act (Liberman and Trope, 2014; Wang et al., 2019). Another common link between the two frameworks is the focus on emotion. SLE explores the idea of formative experiences, which are precisely formative because they are emotionally powerful—for example, hiking for the first time, engaging with animals, and visiting grand vistas in nature (Payne, 1999). Accordingly, in one PD study, deeper emotional engagement diminished psychological distance (Van Boven et al., 2010). In another PD study, researchers found that when people were exposed to an ocean acidification virtual reality experience, their distance from the critters diminished partly because they could empathize with the virtual sea animals (Raja and Carrico, 2021). Moreover, most SLE work examines conservation environmentalists, and less work exists on environmentalists working on climate change (Howell and Allen, 2019). Niche populations like environmentalists are also considerably less examined in the PD literature (for an overview, see Maiella et al., 2020). We use both these frameworks because we think formative experiences and how perceptual distance or its absence influences contemporary environmentalists is an open and unanswered question that, when investigated as we do here, may provide a more layered understanding of the factors motivating this group.

Environmental engagement variables are measured in various forms within the psychological distance and broader engagement literature, for example, policy support (Shwom et al., 2008), risk perception (Van Der Linden, 2015), youth climate activism (Fisher, 2019), and environmental concern (Spence et al., 2012). What is the relationship between psychological distance and environmental engagement? Psychological distance and engagement are generally negatively associated (Spence and Pidgeon, 2010; Jones et al., 2017; Loy and Spence, 2020). Specifically, causal evidence shows that greater psychological distance reduced intent to engage in pro-environmental behaviors (Jones et al., 2017). Other causal research, though, fails to find evidence of a relationship between lower psychological distance and measures of engagement (Brügger et al., 2015; Brügger, 2020), for example, policy support (Shwom et al., 2008).

These mixed findings suggest a need for more research to understand the relationship between psychological distance and environmental engagement (Spence et al., 2012) especially given the complex nature of what diminishing psychological distance to climate change and environmental problems means for individuals (Brügger et al., 2015). We also note that evidence supports a trend suggesting a negative association between psychological distance and environmental engagement (Spence et al., 2012; Jones et al., 2017). It should follow that when we examine the motivational tapestries of environmental engagement as relayed by environmentalists, descriptions of low psychological distance to climate change and other environmental problems should emerge in their life stories. However, this postulate has received little consideration within the psychological distance literature. As such, in this study, we look at this understudied population—environmentalists and their psychological distance perceptions of climate change and other environmental problems. Research suggests that the PD subdimensions are related to each other (Fiedler et al., 2012), so we expect PD subdimensions to parallel PD trends.

Furthermore, much of this research also surveys people from the general public (Spence et al., 2012). Missing from the psychological distance literature is attention to how psychological distance operated in the lives of understudied populations like environmentalists. Because environmentalists are distinct by the amount, type, and ongoing nature of their environmental engagement, it is possible that findings from studies that survey the general public on psychological distance and consider other measures of environmental engagement may not apply here.

### 2.3. Limitations of existing work and our contributions

We underscore several limitations of the existing work discussed above. First, this research is often focused on a narrow definition of who an engaged person is (Tanner, 1980; Chawla, 1999; Williams and Chawla, 2016) to the exclusion of other types of environmentalists, for example, those that engaged in activism, especially on climate change. Our sample comprises environmentalists from diverse backgrounds and those who engage differently. For example, some environmentalists work on environmental protection issues full-time, whereas others volunteer on climate change campaigns. Third, our examination seeks to determine if the formative experiences described within the SLE work still hold for contemporary environmentalists in our sample. Finally, we seek to apply the psychological distance literature (Spence et al., 2012) to our sample of environmentalists, rarely examined in this literature. In so doing, we aim to deepen our understanding of how environmentalists interact with psychological distance and overcome the apathy associated with it.

This inquiry uses qualitative methods, which are well-equipped to investigate formative experiences for three reasons (Chawla, 2006). First, the participant shares a life-span perspective they relay in their words, gestures, manner, and tone. Participants’ voices, stories, thoughts, feelings, and experiences are thus at the forefront of this study. Second, qualitative research is helpful for constructs that are difficult to measure quantitatively, like subjective experiences. This research may clarify the relationship between formative experience, psychological distance, and environmental engagement for a typically understudied population.

To frame this inquiry, we ask two overarching research questions: (1) What formative experiences drove environmentalists to engage in the issue of climate change and environmental protection? And (2) What role (if any) does psychological distance play in the reflection stories of environmentalists on their pathway to engagement?

## 3. Methods

### 3.1. Participants and recruitment

We conducted 33 semi-structured interviews between July 2019 and April 2020 in Boulder. Interviews occurred in person, via telephone, or through *Zoom*, allowing us to widen the scope to environmentalists outside of Boulder, only 11 out of 33 participants were from Boulder (Table 1). Researchers typically recommend between 6–15 interviews and up to 200 excerpts for thematic analysis (Braun and Clark, 2013) to properly uncover patterns. Participants were recruited using purposeful and snowball sampling from our existing social and professional networks. Purposeful sampling allows researchers to specify specific criteria (Patton, 2002). We recruited environmentalists by emailing people who met three criteria: (1) held the basic premise of climate change to be true and urgent; (2) identified as being actively engaged in environmental/climate change advocacy efforts for at least 5 hours in the previous 4 weeks; note: this included people who volunteer, who advocate, who work in environmental professions, for example, science communicators, journalists, museum professionals, and who engage in climate change or environmental protection focused research; (3) were at least 18 years of age. We asked these people if they knew any environmentalists who might want to participate in this study. Interview participants received no incentive for their participation. For a detailed look at participant demographics, please see Table 1. The Institutional Review Board approved the research of the University of Colorado under Protocol 19-0458.

**Table 1:** Socio-demographic indicators of study 1 respondents (*n* = 33).

Variable	Sub-category	^*^Freq.	%
Gender	Female	25	76%
	Male	8	24%
Race	White	25	76%
	Mixed Race	4	12%
	Hispanic	2	6%
	Black	2	6%
Age	Mean	41	
	Minimum	20	
	Maximum	76	
	Standard Deviation	15.9	
Politics	Liberal	32	97%
	Moderate	1	3%
Occupation or Field	Academia	12	36%
	(2 undergraduates|9 PhDs|1 Professor)		
	Attorney	2	6%
	Engineer	1	3%
	For-Profit Consultant	4	12%
	Non-Profit Worker	9	27%
	Teacher^**^	5	15%
Paid or Volunteer Environmentalists Status^***^	Formal Paid environmentalists	12	36%
	Volunteer Unpaid environmentalists	21	64%
Education attainment (Highest level attained)	High School	2	6%
	Bachelors	8	24%
	Masters	9	27%
	J.D.	2	6%
	PH.D	12	36%
Income^****^	Mean	$60,081	
	Minimum	$7,000	
	Maximum	$225,000	
	Standard Deviation	$47,703	
Monthly hours engaged	Mean	91 h	
	Minimum	10 h	
	Maximum	160 h	
	Standard Deviation	60 h	
Home state^*****^	Colorado	11	33%
% Married	Yes	17	52%
% with Kids	Yes	5	15%
% with Pets	Yes	10	30%

^*^Count unless otherwise indicated. ^**^Two persons were retired but listed teacher as their primary occupation before retirement. ^***^Volunteer designation was assigned only in cases where the person was not compensated for their work on climate change or other environmental issues-excludes meals provided at events. ^****^One person did not report income. ^*****^Does not add up to 33 because one participant identified a country outside the U.S. as their home site.

### 3.2. Data collection

In a semi-structured interview, we asked questions about three core themes: (1) formative experiences, including key experiences that explain why people became involved in climate change or other environmental protection work; (2) self-perceptions about psychological distance to climate change and other environmental issues; (3) demographic questions. We asked probing questions for the first two themes (see protocol in Supplementary materials). Participants were given a consent form describing the study before the interview and have its audio recorded. Participants were then asked a series of demographic questions. Interviews then began with broad probing questions such as, “How did you become involved in the issue of climate change or other environmental protection work?” “What was the moment or series of moments that crystallized your involvement in this work?” Interviews ranged from 38 min to 97 min. When the interview no longer yielded new information, in other words, the saturation point was reached, data collection concluded. Please note that pilot interviews with seven environmentalists were first conducted, which helped researchers adjust the research design.

### 3.3. Analytical approach

We applied thematic analysis to our data because of this method’s “recursive” and flexible nature (Braun and Clarke, 2006, 2012, 2021). We drew on Braun and Clarke’s (2006) six recommendations for thematic analysis. First, we transcribed the data corpus of all the interviews from our respondents. The first author read the data closely to become familiar with it. In the second phase, we generated the initial universe of codes. In the third stage, we began to search for reoccurring themes. In the fourth stage, we reviewed the coding terms to see if they fit the data well. In the fifth stage, we finalized the codes and came up with clear definitions. We then went to our data corpus in the sixth stage to identify salient examples to illustrate the codes we came up with. For both questions, we used an essentialist or realist method—which assumes that the participants relay their own experiences and meanings; we did not do a discursive analysis because we were not looking at the social meanings of the data but rather what the participants were relaying to us in their own words (Braun and Clarke, 2006).

To uncover the formative experiences driving environmentalists (RQ1). We used inductive thematic coding because we wanted the environmentalists to tell us what they felt was most important in their engagement story. We used deductive thematic coding to understand the role of psychological distance as a possible driving force propelling environmentalists to act (RQ2). We developed *a priori* codes to correspond with the four subdimensions of PD found in the literature (Spence et al., 2012). We first coded excerpts that demonstrated a participant discussing their psychological distance to climate change and other environmental problems. We also looked at instances of psychological closeness to environment problems, coded when psychological distance was absent. We used both semantic and latent coding. Semantic coding occurs on the surface, for instance, how many times a word repeats. For example, we looked at how many times people mentioned nature experiences. Latent coding uncovers respondents’ hidden assumptions and beliefs by identifying reoccurring themes and patterns in the data (Terry et al., 2017). For example, when participants spoke about traveling, some simply listed all the places where they had been. In contrast, others mentioned several positive feelings about a place, like “intense happiness” or “feeling a sense of warmth.” The participants did not explicitly say place attachment, but these emotional descriptions led the researchers to conclude an underlying concept of place attachment that came through in the coding process.

Moreover, thematic analysis traditionally does not require intercoder reliability but is a flexible method that can be adapted to meet the objectives and reflect the study context (Braun and Clarke, 2006, 2012, 2021). We use a second coder to augment the thematic coding for two reasons. First, we wanted to make sure different researchers would agree on the interpretation of codes. Second, Chawla (1998) observes that one of the weaknesses of SLE research is that it often lacks inter-coder reliability. Two independent researchers coded this study’s data over eight iterations to ensure reliability. Coders agreed on 90 percent of codes in Phase 1. In Phase 2, coders discussed disagreements by providing their code interpretation. Afterward, the coders reconciled their disagreements with a 100% agreement rate. An intercoder agreement of 85 percent is considered highly desirable (Miles et al., 2014). We used *Dedoose* to perform data cleaning and analysis.

## 4. Results

Analysis revealed seven unique themes in the data: (1) nature influences: experience in nature and connection with nature; (2) formal educational experiences; (3) role models; (4) prior civic activism; (5) place attachment; (6) travel experiences, and (7) trauma: general trauma and environmental trauma. Please see Tables 2, 3 for a detailed look at code definitions.

**Table 2:** Formative influence codes identified by the highly engaged.

Theme	Description	Example of excerpt
1. Nature Influences		
Experiences in Nature	Time spent in nature, or engaging with nature, often involves physical activity in nature: fishing, hiking, camping, etc.	“When I was young, my parents would take us fishing and camping in the mountains.”
Connection with Nature	(1) Feeling positive, (2) escape from the mundane, often the burdens of everyday life, (3) having a sense of connection with something bigger (the universe, nature, God) than oneself, (4) flow or being lost in the current moment and, experience timelessness (Williams and Harvey, 2001).	“Going into nature replenishes me and makes me feel calm.”
2. Formal Educational Experiences	Learning about environmental threats, especially climate change in a formal environment: such as schools, museums, and camps.	“I had the opportunity to learn about climate change by doing an independent research project in college.”
3. Role Models	Influences that motivated participants to do environmental protection work. Family, friends, and educators were all mentioned. Some participants mentioned ideas: i.e., religion influenced me.	“I became interested in this work when a teacher in high school mentored me.”
4. Prior Civic Activism	Previous exposure to volunteering or working on other civic issues, for example, protesting the Vietnam War and helping close down nuclear power plants. This could also apply to someone in the participant’s inner circle, i.e., a highly engaged parent or partner whose civic activism motivated the participant also to act.	“I was the youngest protester going with my parents to close down a nuclear power project that probably had some impact on me.”
5. Place Attachment	Emotional connection or speaking of some bond, special meaning to a physical place or site.	“Vising the New Jersey shore was an intense experience; that is where we had spent our summer vacations; it was where I had learned to love nature.”
6. Travel Experiences	Different worldviews or questioning brought on by traveling outside of one’s comfort zone. Traveling to other locations beyond where the person lives.	“My work experiences in Nicaragua informed me how the other half is living.” “I am very well traveled, and travel regularly and so have a global worldview.”
7. Trauma		
General Trauma	Previous traumatic experiences (bad feelings came up) could occur for example through a loss of a family member, physical assault, bullying, etc.. Or growing up with a traumatic event in the backdrop—i.e. my parents divorced. (DSMV 5)	“My high school sweetheart was killed by the Vietnam War; I became aware of loss.”
Environmental Trauma	Participants speak about harm, and injury to the environment, which is the focus, and how this has harmed them personally, physically, or emotionally (often-long lasting).	“Every day I think about climate change and it causes me intense stress and grief. Sometimes I do not feel like getting out of bed”

**Table 3:** Formative experiences frequency by participants (*n* = 33) and mentions (*n* = 744).

Formative Experiences	Number of participants	% of entire sample^**^	Total mentions^**^	% of mentions
1. Nature Influences				
Experiences in Nature *of which*	30	91%	185	25%
*As a child*	27	90%	111	60%
*As an adult*	13	43%	33	18%
Connection with Nature	23	70%	65	9%
2. Formal Educational Experiences	26	79%	49	7%
3. Role Models	26	79%	92	12%
4. Prior Civic Activism	23	70%	26	3%
5. Place Attachment	18	55%	84	11%
6. Travel Experiences	16	48%	58	8%
7. Trauma				
Trauma				
General Trauma	22	67%	25	3%
Environmental Trauma	20	61%	70	9%

^**^Total is more than total *n* because participants mentioned more than one formative experience.

### 4.1. Formative experiences of the highly engaged (RQ1)

The nature theme was mentioned most. It is broken into two distinct themes, experiences in nature and connection with nature. Ninety-one percent of participants referenced experiences in nature, of whom 90% alluded to experiences in nature as a child, and 43% alluded to experiences in nature as an adult. Participants further mentioned involvement in multiple physical activities such as hiking, fishing, camping, and gardening, often for prolonged periods (30 day wilderness trips and overnight hikes, for example.) One person remarked how lucky she was because there was a pond on her family’s property. Others spoke of growing older and making sure to “make time for nature” and imparting the importance of time spent in nature to children.

Friends and family were frequently a part of experiences in nature. One participant spoke of hiking with her mom and picking wildflowers as an annual ritual that was a cornerstone memory of her childhood. Another talked of birdwatching with his grandmother as “some of my happiest memories.” Access to experiences in what they called “pristine” nature was important to participants. Many spoke of having access to isolation in nature where they could “escape” and “think.” Others in more urban locations (New York City and Chicago, for example) spoke of their experiences in nature as encompassing going to the local park or the zoo but emphasized that they made sure this occurred on a routine basis. One person spoke of an “ever-present” need to be in nature, which led her to construct a greenhouse in her backyard.

Seventy percent of the overall sample referenced a *connection to nature*. In contrast to experiences in nature, these excerpts often referred to the emotions and feelings that came up for participants when they spent time in nature. Environmentalists reported feeling connected to nature when they felt positive feelings, for example, when they felt “calm and like at home,” and used terms like “replenishment,” “escape,” “solace,” “excitement,” “rebirth,” “serenity.” Others alluded to a “sense of spirituality” acquired only in nature. Some expressed the sentiment that they “were in awe” of nature, felt insignificant in comparison to nature, felt “less than an insect,” and were “humbled by its capacity to endure.” Many participants also humanized nature. One described it as “nature was a living, breathing beautiful [entity.].” When reflecting on climate change, one person remarked it is as if “Mother Nature, she has a fever, and I have to do something about it.”

Of note, environmentalists of color in our sample often described being in nature as a collective rather than an isolated activity. For example, one Black environmentalist said, “Black people like to participate in [nature activities] that are not necessarily classified as legitimate outdoor love for recreation type[s], or I would say mainstream activities. Right. And so [we connect to nature via] family reunions, [which is a thing] for especially African Americans, [where] the outdoors is a place to connect with people.”

Seventy-nine percent of the sample alluded to *formal educational experiences*. Many people spoke of coming to an awareness that the environment “was in trouble;” often, this occurred in a formal educational environment (for example, schools, museums, and camps), which acted as a gateway sparking further interest. Many said they were unaware of climate change until high school or college. Others spoke of direct exposure to nature and her inhabitants and feeling impressed by them. The example below demonstrates a participant’s love for the environment and how this exposure made him respect nature:

When I was 13, I did a summer program. It was a free program in this rural part of Virginia...one of the things that we did as part of this field experience we collected salamanders. And I don't know if you've ever tried to collect salamanders, but they're not easy to find, really hard to catch. So, we spent two days on the side of the mountain, on different elevations on the side of the mountain catching, identifying salamanders...as the day got longer and the salamanders got harder to catch. I was very impressed by nature's ability to put up a fight.

Seventy-nine percent of the sample referenced *role models*. Parents, teachers, grandparents, and friends were most referenced. Participants spoke of how the role model taught them to “care” and “respect” the environment. For example, one participant reflected: “My grandpa [was] the gardener in his home. And I think even from a very early age, [he taught me] reverence…for the idea of the relationship between me and the garden.” Another participant recalled a vivid memory of camping with her dad: “There was this birch tree, and I was bored, and I just went over to [it] and like started peeling off the bark, and my dad freaked out on me, and he was like so intense. And he’s like, never do that. That is the way the tree protects itself. You’re like taking away, you know, that’d be like peeling off your skin.” Others spoke of public role models who influenced them like Jane Goodall, Van Jones, Steve Irwin, David Attenborough, or Al Gore. Participants also mentioned influential books they valued: *Island of the Blue Dolphins*, *Julie of the Wolves*, and *The Lord of the Flies*. Other motivating influences were belief systems, including religion. One participant remarked, “A part of the philosophy that my family follows is Japanese-related. And one of the pillars of it is related to like paradise on earth…this paradise on earth is based around beautiful nature…[and] being appreciative of nature.” Others also spoke of how their interpretation of a particular belief system (Buddhism and Shintoism, for example) all teach stewardship towards the earth and “her” creatures.

Seventy percent of the sample referenced *prior civic activism*. Participants in this category had worked actively on a social problem. Many protested when they were younger (Vietnam and Iraq War, for example), while others said their parents were very active in their local communities, which rubbed off on them. Some participants mentioned parents taking them to protests as a child. Participants attributed these prior civic exposures to their need to act. Nearly all the participants were founding members of organizations, legislative efforts, policy documents, and local campaigns (shutting down a coal plant, for example) relating to climate change or other environmental protection issues. Others spoke of environmental service work (clean water, recycling, renewable energy) done as children or adults, often abroad, and other volunteer efforts with groups combatting climate change or other environmental threats.

Fifty-five percent of the sample referenced *place attachment*. Participants in the sample spoke of an emotional connection to a place and were concerned that the places they had bonded with were changing and, in some cases, even disappearing because of climate change. Many participants spoke of sites with special meaning to them or where they grew up. Others told of “being unable” to leave a place because their parents, children, and friends were all there, so they “had no choice but to act on climate change.”

Forty-eight percent of the sample referenced *travel experiences*. Of the 48 percent, nearly half spoke of traveling to different places, places that forced them “out of their comfort zone;” one person described herself as a “global citizen.” Most of the travel experiences participants mentioned were international. Some reflected that travel experiences helped them connect environmental change to other phenomena. For example, one participant spoke of traveling and working in Nicaragua and how this work “showed me the tie between poverty and environmental changes.” Another participant remarked, “The turning point came for me. I was in Bolivia in 2010 for working on a public health project there and saw firsthand the amount of glacier melt on the Andes.” Other people spoke more about this “witness status of environmental change” by reflecting on coral die-off, tree line shifts in the polar regions, and poor air quality in Mexico, Indonesia, and China due to heavy industrial pollution. One participant spoke of feeling “guilty” about creating a large carbon footprint because of all the traveling he had to do.

Sixty-seven percent of the sample reflected on a *traumatic experience* when deciding to do environmental protection work. Some made this link explicitly, while others hinted at the connection. Most participants spoke of the loss of close people and how this familiarized them with loss. One person stated: “My high school sweetheart was killed by the Vietnam War; I became aware of the loss.” He then spoke of the Vietnam War as a “human disaster” and linked it to climate change as “another human disaster.” Many participants recalled high school experiences of feeling bullied. One reflected that after she was bullied, she did not “want the earth to feel that.” One participant spoke of feeling “abandoned” when her parents divorced and “could not imagine how the planet deals with that.” One participant spoke of being involved in “a physical assault with a partner” and how “she felt powerless having no voice.” This difficult experience “made her empathize with how the Earth feels.” Another participant spoke of being homeless and living in her car. Another, the only one who became emotionally upset, said she had lost “a number of relatives who have contracted cancer in ways that I’m pretty sure are due to pollution.”

Many participants linked personal trauma to healing in nature. They only revealed they had experienced trauma by first talking about the process of healing from it in nature. For example, some spoke of the healing potential of the natural environment. Others described it as “healing in nature.” One person said it was “so important… [because] my family life was not joyful. It was not a wonderful place to be.” Another remarked, “In college, I used to get into a lot of trouble, and that was a dark time (participant was physically assaulted) for me, and so I would run to nature to escape and reset.” Another participant reflected: “I lost a very close family member, who had introduced me to nature, and that really hurt.” One participant remarked: “I had lost one of my girlfriends to suicide…and…so, I found a lot of solace in going [to]the natural world.” Another participant was in a “violent car accident” and went to nature to “heal.”

Sixty-one percent of the overall sample referenced environmental trauma, specifically. Environmental trauma was differentiated from general trauma in three ways. First, the initial harm was directed to the environment. Second, a person experiences harm only from exposure to environmental protection work. And third, a person experienced harm in the environment. For example, some spoke of how witnessing this “climate change travesty” has caused them physical and emotional damage, often-long lasting. One participant spoke about how “heart-wrenching seeing the environments I love dying in front of my eyes [has been].” Many in this category spoke of the emotional and physical toll doing environmental protection work takes, including debilitating stress and anxiety. Others spoke of being chronically depressed by doing environmental protection work because of witnessing the “deep abuse” the environment suffers. One participant remarked:

[I] grieve, almost all the time [about climate change]…and hurt to the planet…there have been years when I don’t sleep well, and that's, you know because it’s not going to be my kids’ problem… it’s part of the reason why I don’t have kids…This is…a dying world for them to be in and their kids, and this decision has been very traumatic to arrive at.

Excerpts in which environmentalists spoke about the personal toll they had experienced due to working in the environmental protection arena were also coded as environmental trauma. For example, one participant spoke about the consequences of doing environmental protection work: “I had [powerful interests] trying to destroy my life. That got a little more stressful…I was at a doctor’s appointment. They noticed I had an irregular heartbeat… and I had lost a lot of weight. So, there was, I would say, there was a health toll… [because] the stress was getting so bad.” Another participant talked about needing to get “back on Prozac” because of the toll the work was taking. Another climate change activist spoke about going “broke and bankrupt,” doing this work. Another said the emotion she attached to climate work was “intense sadness for sure.” Another stated: “You realize that everything you have trained for is not enough to stop the destruction.”

Others spoke about the dangers of doing environmental protection work, as illustrated by the example below:

I have heard a lot about people getting murdered because they are like hugging a tree…[or] trying to stop…[particular interests] from invad[ing] particular areas. There are protected areas for several different ethnic groups…environmentalists come in, and they’re trying to support the community as well as nature. Right. And then they get killed…

Excerpts in which environmentalists experienced trauma in nature were also coded as environmental trauma. For example, one Black participant spoke about how nature became the site of trauma. She reflected on a physical attack she experienced: “I was walking in the woods, hiking with my dog, and I was…physically assault[ed], by that I mean two white women who did not like the color of my skin, set the dog on me. and said as much.”

She also made the following observation about the symbolic meaning of the woods to her:

A lot of African Americans are afraid of the woods… it’s because…of our history of lynching and slavery…Things can be done to you in the woods…because of the anonymity…nobody can see what is happening. People can do things to you anonymously and get away with it…. There’s a long history of violence against Black people in wooded areas and that's why we often avoid the woods…. It took me a while to return to the woods.

### 4.2. Role of psychological distance environmentalists engagement (RQ2)

**Figure 1:**
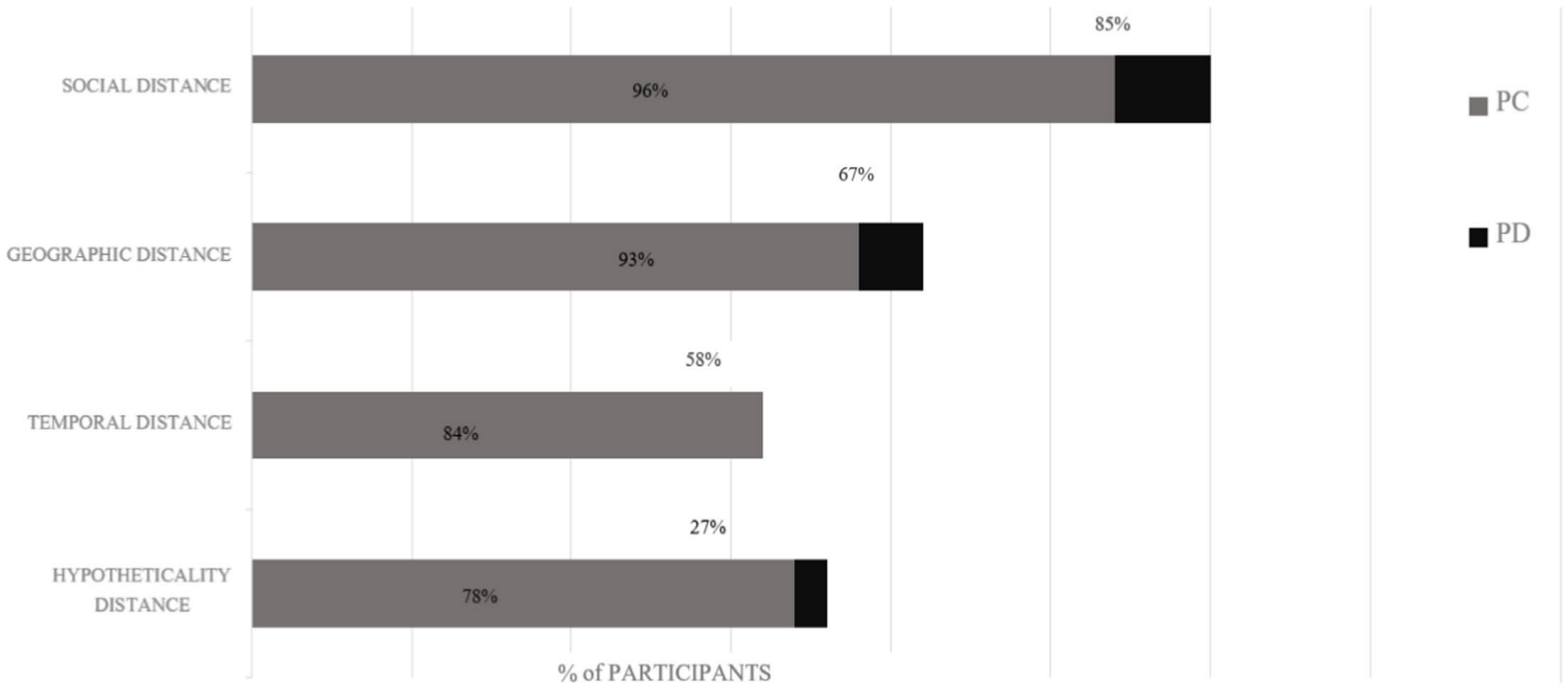
P.D. mentions and the P.C. breakdowns by subdimension for participants. The first number from the right alludes to P.D. The second number closest to the left indicates P.C. For example, 85% of participants mentioned social distance P.D., of whom 96% expressed feeling low social distance or P.C. Percentages add to more than 100% because some participants expressed both sentiments. The length of the entire bar ranks subdimensions. For example, participants spoke about the social distance the most and hypotheticality the least.

Our second objective was to understand how environmentalists spoke about the psychological distance between them and environmental threats and whether perceptions of distance appeared to be linked to the decision to engage. We find that at least one psychological distance dimension was mentioned by 91% of respondents. Of these mentions, 94% expressed closeness (i.e., the absence of P.D.) to climate change on at least one P.D. dimension (Figure 1). Below, we discuss the four sub-dimensions of P.D.

*Social P.D.* was the most mentioned subdimension. Eighty-five percent of environmentalists alluded to it, of whom 96% felt socially close to environmental problems (vs. distant; Figure 1). environmentalists referred to feelings of closeness when they discussed that their family or friends were experiencing or had experienced environmental problems. They spoke about this direct experience with the following environmental problems: wildfires, drought, hurricanes, health-related environmental issues, and environmental justice issues. Many participants also said that seeing their loved ones on the front lines of environmental problems made them feel as if they were “bound to environmental work.” Referring to this obligation, one participant remarked, “My parents are in their eighties and I kind of feel like I cannot leave cause…they live in Colorado and...they are most impacted by wildfires...and so I do not really feel like I could leave [this work].”

Environmentalists also said that they felt that something was “wrong.” They said they had spent so much time in nature. After a while, they began to notice changes in their environment, which signaled that something was “very wrong.” The following example from a volunteer environmentalists demonstrates how this proximity to nature brought them closer to environmental degradation.

We were highly involved in going to the farm, eating things that were planted in harmony with the soil….We were always exposed to nature, [and then when we went to the farm] the soil is dryer…we…[are] noticing changes.... The flowers are blooming later…. That was something that I was exposed to a lot and that helped me get engaged with thinking and understanding climate change.

*Geographic P.D.* was the second most alluded to subdimension. Sixty-seven percent of participants mentioned geographic P.D., of whom 93% expressed feelings of geographical closeness to environmental problems. Environmentalists referred to these feelings and thoughts in one of two ways. First, they spoke of being physically close to an environmental problem. For example, one environmentalists reflected on growing up and being “truly in love” with Southern California’s water bodies and then observed that “seeing the natural systems that I truly love become altered has been really transformational and heart wrenching; seeing the environments I love dying in front of my eyes.” The second way closeness to environmental problems manifested was that some environmentalists spoke of the fact that despite the physical distance from an environmental problem, they perceived it as close because they had some connection to it. This sentiment frequently arose for environmentalists who had personal links to a physical place. One environmentalists reflected on how Hurricane Maria decimated her family home in Puerto Rico in 2017. Even though she did not have any family living there, she still cared about the damage Puerto Rico had suffered and was worried how similar future events might wreak havoc on the island.

*Temporal P.D.* was the third alluded to subdimension. Fifty-eight percent of environmentalists mentioned temporal P.D., of whom 84% expressed feelings of temporal closeness to environmental problems. No one expressed feelings of distance on this issue (Figure 1). Temporal dimension mentions took one of two forms. First, environmentalists spoke about how the environmental problems around them changed over time. For example, one participant remarking on the bigger issue of climate change said, “I’m noticing...violent...weather pattern changes in the places that I’ve lived in over the last decade… more wildfires in this area than before and realizing how that’s going to get worse.” Second, environmentalists spoke about the future consequences if a particular environmental problem remained unaddressed. Several environmentalists also attached the word “urgency” to their remarks about the future. Many environmentalists also noted that they wanted to leave the planet in “better shape for their kids...as the future was...[already]...looking bleak” for future generations.

*Hypotheticality P.D.* was the least alluded to subdimension. Twenty-seven percent of participants mentioned hypotheticality, of whom 78% expressed feelings of psychological closeness (Figure 1). Participants spoke about hypotheticality in relation to how acquiring more personal knowledge about environmental problems, chief among them climate change, made them decide to “jump off the fence” and commit their efforts to direct action. Moreover, older environmentalists reported that climate change was a relatively “new term” for them, and through talking, learning, and seeing how “horrific [climate change] was and could be,” their belief in the issue’s importance was reinforced. How this subdimension emerged suggested that most participants, especially older environmentalists, felt uncertain about climate change only when they did not have enough knowledge on the subject.

## 5. Discussion

### 5.1. Nature experiences remain salient for contemporary environmentalists

Nature influences were the most formative theme that environmentalists reported. Moreover, the SLE literature features it prominently (Tanner, 1980; Hines et al., 1987; Chawla and Flanders Cushing, 2007). Interestingly, many environmentalists from urban areas also spoke of camp activities or fellowship opportunities that allowed them to experience pristine nature, which ignited their desire to “bottle” those experiences and bring them back to the city.

Connection with nature emerged as a distinct type of influence slightly different in character from experiences in nature. Specifically, environmentalists spoke of connecting to nature as more meaningful by focusing on the emotions they felt. Within the literature, less is known about *why* some experiences in nature are meaningful, and others are not. Among the few researchers to explore the precise question of why this connection with nature is so powerful are Williams and Harvey (2001). They surveyed 131 people who frequently go to forests about their thoughts and feelings and found that people most affected by the forest experienced what the authors term a “transcendent experience.” A person may identify feeling transcendent when they experience an abundance of positive feelings in nature, a sense of escape from the burdens of everyday life, or when they experience a sense of connection with something bigger than themselves—for example, the universe, Mother Nature, or God (Williams and Harvey, 2001).

We saw evidence of this transcendence in our environmentalists, who touched on all these aspects of connection with nature. We suggest that powerful transcendent connections with nature, especially earlier in life, maybe incredibly formative for those who become highly engaged. For instance, environmentalists turned to nature to escape as a spiritual act and, in some cases, as an act of rebellion. To illustrate, one participant spoke of skipping school to immerse herself in nature throughout her high school years. Many of these environmentalists also focused on how they felt and the emotions that came up, for example, feeling happy, content, peaceful, and in awe when they felt deeply linked to nature.

Trauma was an unexpected theme that emerged from our interviews. Some SLE work has examined young people’s grief experiences with the loss of a place. For example, Fisher (2016) interviewed 17 youth activists and found that participants felt a profound loss when they saw landscapes disappear. One activist spoke of feeling depressed after seeing the Pokhara mountain in Nepal. He was expecting to see Pokhara snowcapped, but instead, there was no snow. Other work in this vein also details the negative effects on children when they view the destruction of natural areas (Chawla, 1999, 2020; Chawla and Derr, 2012; D’Amore and Chawla, 2020). In one study, children were visibly affected. Many cried when the trees and their playground were bulldozed (Blizard and Schuster, 2004). Much of this research examines the loss felt by youth. Another commonality of this research is that it mainly looks at place-based loss. Additional research on a more expansive definition of loss and trauma is needed, especially since environmental destruction is increasing.

Our study sample discussed place-based and personal trauma encounters as influencing their engagement. For example, they spoke about habitat destruction experiences and how other more personal traumas drive their need to engage. For instance, participants spoke about a loss of a loved one or experienced battling a severe illness and spoke about how these experiences made them empathize with Mother Nature and the “harm she goes through.” Moreover, participants did not speak of one event of salience that stood out but multiple, often intersecting events. For example, one participant mentioned growing up in an unstable home and facing a bully at work. Other work should explore these intersecting layers of trauma and how they collide to affect engagement choices.

Sixty-seven percent of the sample reflected on a traumatic experience that influenced their engagement. Trauma was most often experienced directly. Such trauma took the form of physical violence, intimidation, violation of one’s intimate or physical space, and a lack of security, i.e., enduring being homeless. We also saw an association between someone explaining how it felt to be the recipient of harm and a feeling of empathy for the planet. For example, many spoke of “how it felt” to be “bullied, voiceless, abandoned” and compared it to how the earth feels.

While many of the environmentalists we interviewed spoke about going into nature to heal from personal trauma, others—particularly environmentalists of color—said that nature was a stage for trauma. For example, two Black environmentalists spoke about the fear of personal safety in wooded areas or on trails. One Black environmentalist spoke of a racially motivated physical attack in this space. These findings support scholarship that finds that prejudice and discriminatory acts could make people of color feel unsafe and unwelcome in green spaces (Gobster, 1998; Byrne and Wolch, 2009). One participant who experienced this act of hostility said it took some time for her to return to the green space in which the act occurred. Recent campaigns that have sought to make green spaces more “inclusive” (Ernst, 2021) and safe should consider that some participants could be viewing that green space as a place of trauma, which could be altering how they interact with that green space.

We further found evidence of two specific strains of trauma, one that was general and one that was explicitly environmentally in nature. In the latter camp, environmentalists spoke of experiencing environmental trauma due to being on the frontlines and of their intense sorrow at observing environmental catastrophe. There is increasing recognition that environmental protectors work within disappearing ecosystems and with species and landscapes under threat in a biosphere that is “irreparably damaged.” In Aldo Leopold’s words (1954), they exist in a “world of wounds” while continuing to fight every day on the front lines of climate change, biodiversity loss, landscape restoration, wildlife trafficking, or other complex environmental issues (Ojala, 2015). Other researchers like Cunsolo and Ellis (2018) speak about “ecological grief” that is specific to conservation professionals, grief that contributes to personal anxiety over deteriorating ecosystems and environments and is increasing (Clayton and Karazsia, 2020).

We add to this line of work by showing how the highly engaged experience similar sentiments of grief and trauma. Persons in our sample also spoke about physical health ailments ebbing from this work. For example, many spoke about the personal toll of working close to a “dying planet,” physical ailments, lost opportunities, lost careers, and how they had made emotionally heavy and exceedingly difficult decisions. For example, many had decided not to have children because they had experienced the destruction of the environment and apathy toward that destruction. They did not want their kids to enter such a “callous” world or contribute to further destruction. Others provided powerful examples of the health toll of environmental work. Participants spoke of losing weight, losing sleep, taking antidepressants, and experiencing increased blood pressure and irregular heartbeats. This finding may signal a need to develop tailored mental and physical health resources to support the health of the highly engaged and ensure they avoid burnout.

### 5.2. Other SLEs remain salient for contemporary environmentalists

Environmentalists reported other influences as formative. Formal educational experiences, role models, prior civic activism, place attachment, and travel experiences were named. They align with the formative influences identified within SLE work (Tanner, 1980; Chawla, 1998, 1999, 2020). Among these, some warrant further discussion. For example, most of the sample was exposed to prior civic activism. Environmentalists were either involved directly—they had protested a social problem as young adults—or indirectly when their parents took them to protests when they were children. Environmentalists spoke of witnessing such acts of civil disobedience as exposure to nascent forms of social capital, which “taught” them how to mobilize. Scholars have found evidence to suggest that when parents are active in social causes, their children are likely to emulate their behaviors (Chawla, 2001, 2020; Chawla and Flanders Cushing, 2007). We deepen this thread. We find evidence to suggest that exposure to early activism often carried out by parents, teachers, and influential peers provided environmentalists with a repository of knowledge and tools (what one participant referred to as “organizational, social capital”). Seventy percent of our sample of environmentalists emphasized such instances. One environmentalists described them as “training moments.” Interestingly, these environmentalists assumed senior leadership positions, founded organizations/s, and mobilized others on national legislative actions. Such environmentalists thus were not just highly engaged but also highly efficient in the public sphere environmental activism they pursued.

### 5.3. Psychological distance, especially social distance, matters for environmentalists

Our second question sought to understand how environmentalists conceptualized the psychological distance between them and environmental issues and whether such perceptions of distance or absence of (psychological closeness) appeared to be related to their decision to engage. Most environmentalists felt psychologically closer to environmental threats than psychologically distant.

We further find that environmentalists mentioned the social distance subdimension most. Nearly all environmentalists (96%) had either experienced an environmental problem directly or had a family member or loved one in harm’s way. This suggests that the subdimension of social distance may play a vital role for environmentalists. Specifically, for the environmentalists interviewed, direct experience with climate-related harm appears to dimmish psychological distance. For environmentalists, this harm took the form of floods, wildfires, hurricanes, sea-level rise, drought, food-desert issues, and instances of environmental harm induced by poor air quality and rampant industrialization issues close to home. Many participants said witnessing their loved ones experience such events made them feel as if they were “bound” and “shackled” to environmental protection work.

Scholars further suggest that lower psychological distance is associated with higher environmental concern and risk (Uzzell, 2000; Spence et al., 2012). Scholars further suggest that when people experience extreme weather events, their environmental risk perception increases (Van der Linden et al., 2015). Thus, lower psychological distance may be associated with more direct experiences. In addition, Demski and team (2017) compared a sample of residents in the United Kingdom who had experienced a flood with those that did not and found that those that had experienced a flood scored higher on the measures of pro-environmental intent on climate change and support for climate change mitigation policies. We note, though, that other research shows no evidence of an association between experiencing extreme weather events and environmental concern (Demski et al., 2017); however, studies in this vein often sample general rather than highly engaged populations. Consequently, more research is needed on how such adverse environmental experiences shape environmental engagement and the relationship between direct experiences of climate harm and psychological distance. In sum, we show that for environmentalists, direct experiences of climate-related harm suffered directly or via loved ones may be incredibly impactful. Finally, we note that psychological distance could be an understudied formative phenomenon in the lives of environmentalists and warrants further investigation.

## 6. Limitations and future directions

This research is not without limitations. Our qualitative work most likely was impacted by what Chawla (2006) calls the “autobiographical memory” concern. Many participants recalled things that happened in the past and might not be remembering accurately. Moreover, our interview sample was majority female and white and may not reflect all types of environmentalists; a broader and more representative interview sample should be recruited in the next iteration of this research. This limitation is concerning because the literature has paid less attention to how people from different racial and socio-economic backgrounds interact with ever-changing definitions of nature and how this backdrop figures into their decision to be engaged in later life (Tanner, 1998; Matsuba and Pratt, 2013; Molinario et al., 2020). For example, SLE scholars often examine summer nature programs and how these may have shaped an affinity to the environment in later life (Williams and Chawla, 2016). This assumes access to certain financial resources that would have allowed such persons to participate in such opportunities, which may be scarce for those within another income bracket (Hutton and Sepulveda, 2019).

We note that some limitations are common to almost all SLE work. At the same time, our work offers interesting insights into how participants report what they perceive as important in their life stories of engagement. In sum, many formative SLEs still hold weight today compared to when the literature was founded in the 1970s and 80s. The perennial nature of these findings suggests many shared ingredients between contemporary environmentalists and their predecessors. Nonetheless, we underscore the nature and severity of environmental problems has shifted.

## 7. Conclusion

Highly engaged environmental citizens interviewed in this study named nature experiences and connections as instrumental in their decision to engage environmentally. For these, highly engaged connection to nature remains salient in ways consistent with what has been documented in the SLE literature thus far. Our findings also reveal that psychological distance has played a role in the lives of these highly engaged individuals, especially social distance. Finally, a novel contribution of this study is the extent to which the engaged named traumatic experiences as shaping their decision to engage in environmental advocacy, work, and volunteer efforts.

In the future, effective action on environmental problems will be especially contingent on “public action” (Gardner and Stern, 2002). The highly engaged in our sample are active and often public advocates for environmental causes. They are vital in sustaining environmental movements and propelling solutions. As such, we should conduct more research on their formative perceptions and attitudes that could help explain why they have selected action over apathy. We hope the knowledge generated in this exploratory study can help others better understand this often understudied yet vital population.

## Data availability statement

The datasets presented in this article are not readily available to ensure participant confidentiality. Requests to access the datasets should be directed to uraja@luc.edu.

## Ethics statement

The studies involving human participants were reviewed and approved by The University of Colorado at Boulder. The participants provided their written informed consent to participate in this study.

## Author contributions

UR conceptualized the initial study and the research design with input and advice from AC, UR analyzed the data with guidance from AC. UR wrote the article. AC contributed edits and comments during manuscript development. All authors contributed to the article and approved the submitted version.

## Funding

This material is partially based upon work supported by the National Science Foundation Graduate Research Fellowship Program under Grant No. (NSF grant number DGE 1650115). Any opinions, findings, and conclusions, or recommendations expressed in this material are those of the author(s) and do not necessarily refect the views of the National Science Foundation. We thank the Graduate School of Arts and Science at the University of Colorado, the Center to Advance Research and Training in the Social Sciences, and the Department of Environmental Studies for grants at the University of Colorado to offset the cost of data collection. We also thank Kelly Hallisy and the School of Communication at Loyola University for research help.

## Conflict of interest

The authors declare that the research was conducted in the absence of any commercial or financial relationships that could be construed as a potential conflict of interest.

## Publisher’s note

All claims expressed in this article are solely those of the authors and do not necessarily represent those of their affiliated organizations, or those of the publisher, the editors and the reviewers. Any product that may be evaluated in this article, or claim that may be made by its manufacturer, is not guaranteed or endorsed by the publisher.
