# Supplementary material for: Formative experiences and psychological distance in the lives of contemporary environmentalists
Source: Front Psychol. 2023 Jul 18;14:1192018. doi: 10.3389/fpsyg.2023.1192018 (PMC10393244; doi:10.3389/fpsyg.2023.1192018)
Supplement: Supplementary file 1 [file Data_Sheet_1.docx]

**Study – Semi-Structured Interview Guide**

*This study is organized into three sections that correspond to the three themes identified in the research design section. I will probe additional themes as they emerge. Please note some of these themes overlap.*

**Theme 1: Psychological closeness (absence of psychological distance) and its role in HEEC engagement**

1. Can you please tell me what role if any did climate change play in your life? (*Probe*)
2. What was the moment or series of moments that crystallized your involvement in this work? (*Probe*)
3. If you could talk to yourself in 50 years about this work? What would you say and why? (*Probe*)
4. If you were to try to motivate others on the issue of climate change, how would you do it and why? (*Probe*)

**Theme 2:** **Primary Drivers/Motivators propelling HEEC engagement**

1. How did you become involved in the issue of climate change? (*Probe*)
2. Can you please tell me what motivates you to work on the issue of climate change? (*Probe*)
3. Why are you involved in this work? What keeps you going? (*Probe*)
4. Why is this work important to you? (*Probe*)
5. What do you hope to accomplish? (*Probe*)
6. Are you going to keep doing this work? Indefinitely? Do you anticipate a scenario where your future plans do not include engaging in this work? (*Probe*)
7. What would it take to stop you from doing this work? (*Probe*)
8. Could you reflect on a moment or an experience where you felt especially proud of the work you do? Why? (*Probe*)
9. Could you reflect on a moment or an experience where you felt especially disheartened in the work you do? Why? (*Probe*)
10. Who are the people that have been instrumental in motivating you to do this work and why? (*Probe*)
11. Are others in your immediate network as engaged on climate change as you? (*Probe*)
12. What emotions come up when you do this work? (*Probe*)
13. How do you feel (happy, sad, fearful, disheartened, etc.) when you engage in this work? (*Probe*)

**Theme 3: Demographic and Level of Engagement Questions**

1. Which gender do you identify with?
2. Which racial category do you identify with?
3. What is your age?
4. What is your occupation?
5. What is your approximate level of education?
6. What is your estimated annual income?
7. How many hours in a month do you engage in climate change work?
8. Anything else you wish to share? (*Probe*)
9. Are there any questions you feel that I should be asking that I haven’t covered? (*Probe*)

*Final Statement:*

“Thank you very much for participating in this study. Your answers will be kept confidential.

This concludes the interview.”
